# Use-Dependent Prosthesis Training Strengthens Contralateral Hemodynamic Brain Responses in a Young Adult With Upper Limb Reduction Deficiency: A Case Report

**DOI:** 10.3389/fnins.2021.693138

**Published:** 2021-06-11

**Authors:** Jordan A. Borrell, Christopher Copeland, Jessica L. Lukaszek, Kaitlin Fraser, Jorge M. Zuniga

**Affiliations:** ^1^Department of Biomechanics, University of Nebraska at Omaha, Omaha, NE, United States; ^2^Center for Biomedical Rehabilitation and Manufacturing, University of Nebraska at Omaha, Omaha, NE, United States; ^3^School of Pharmacy and Health Professions, Creighton University, Omaha, NE, United States

**Keywords:** hemodynamic response, 3D printed prosthetics, upper limb reduction deficiency, use-dependent plasticity, functional near infrared spectroscopy, motor control

## Abstract

The purpose of the current case study was to determine the influence of an 8-week home intervention training utilizing a partial hand prosthesis on hemodynamic responses of the brain and gross dexterity in a case participant with congenital unilateral upper-limb reduction deficiency (ULD). The case participant (female, 19 years of age) performed a gross manual dexterity task (Box and Block Test) while measuring brain activity (functional near-infrared spectroscopy; fNIRS) before and after an 8-weeks home intervention training. During baseline, there was a broad cortical activation in the ipsilateral sensorimotor cortex and a non-focalized cortical activation in the contralateral hemisphere, which was non-focalized, while performing a gross manual dexterity task using a prosthesis. After the 8-week home intervention training, however, cortical activation shifted to the contralateral motor cortex while cortical activation was diminished in the ipsilateral hemisphere. Specifically, the oxygenated hemodynamics (HbO) responses increased in the medial aspects of the contralateral primary motor and somatosensory cortices. Thus, these results suggest that an 8-week prosthetic home intervention was able to strengthen contralateral connections in this young adult with congenital partial hand reduction. This was supported by the case participant showing after training an increased flexor tone, increased range of motion of the wrist, and decreased times to complete various gross dexterity tasks. Changes in HbO responses due to the home intervention training follow the mechanisms of use-dependent plasticity and further guide the use of prostheses as a rehabilitation strategy for individuals with ULD.

## Introduction

The Center for Disease Control and Prevention (CDC) estimates that about 1 in every 1,900 babies is born with a limb reduction in the United States (CDC, 2020). However, there are many more unreported cases due to lack of a mandatory reporting system of birth defects and child amputees. According to the CDC, the use of an upper-limb prosthesis is one of the main treatments to help restore proper function and appearance in children experiencing upper-limb reduction deficiencies (ULD) (CDC, 2020). For children with ULD, the use of prostheses is directly related to the success of rehabilitation outcomes including development of motor skills, performance of activities of daily living and recreational activities, and improvements in self-esteem (Zuniga et al., 2015, 2016, 2019a; CDC, 2020).

Previous literature suggest the involvement of specific neural control mechanisms that limit the functional use of these devices during initial prosthetic usage which could contribute to the high rejection rate of prosthesis users (Meurs et al., 2006; Huizing et al., 2010; Hadders-Algra et al., 2013). However, there is a lack of data showing the neural mechanism of novel rehabilitation approaches in the pediatric population (Hadders-Algra et al., 2013; Hadders-Algra, 2018). Using transcranial magnetic stimulation (TMS), Reilly and Sirigu suggest that subjects with congenital absence of a limb do not develop representation of the absent limb within the motor cortex (Reilly and Sirigu, 2011), which supported an earlier case study (Cohen et al., 1991). Instead, the missing hand/limb region becomes utilized by its cortical neighbor, depending on the compensatory strategy of other muscles and body parts (i.e., use-dependent plasticity) (Makin et al., 2013; Hahamy et al., 2015, 2017).

In a previous investigation, our research team showed that a 6-month use of a wrist-driven 3D printed partial hand prosthesis significantly lowered the coactivation index of the wrist flexors/extensors by 70% in ULD children, indicating an improvement in motor control strategies of the affected limb (Zuniga et al., 2018). More recently, our research group found an abnormal activation in the ipsilateral motor cortex in ULD children while using an upper limb prosthesis during a gross manual dexterity task (Zuniga et al., 2021). Previous investigations have shown that adults with ULD experience significant use-dependence plasticity after increased usage of the residual limb (Makin et al., 2013; Hahamy et al., 2017). Specifically, it has been shown that people with congenital hand absence, who are better at incorporating their residual arms in daily tasks, activate their missing hand cortex by whichever limb individuals are over-using during daily tasks (Makin et al., 2013). It was further shown that the missing hand territory can be inhabited by compensatory behaviors that do not cortically neighbor the hand territory (Hahamy et al., 2017). However, it is uncertain if this use-dependent plasticity occurs after the use of a partial hand prosthesis or if children with ULD will show a similar response to adults. Based on previous literature (Makin et al., 2013; Zuniga et al., 2021), it was hypothesized that, (i) before prosthetic usage, there will be an broad ipsilateral control of the affected hand using the prosthesis, (ii) after an 8-week home intervention training performing unimanual and bimanual activities using a partial hand prosthesis, use-dependent plasticity will occur resulting in a more prominent cortical activation in the contralateral hemisphere while using the prosthesis.

## Case Description

### Case Participant & Anthropomorphic Measurements

**Figure 1:**
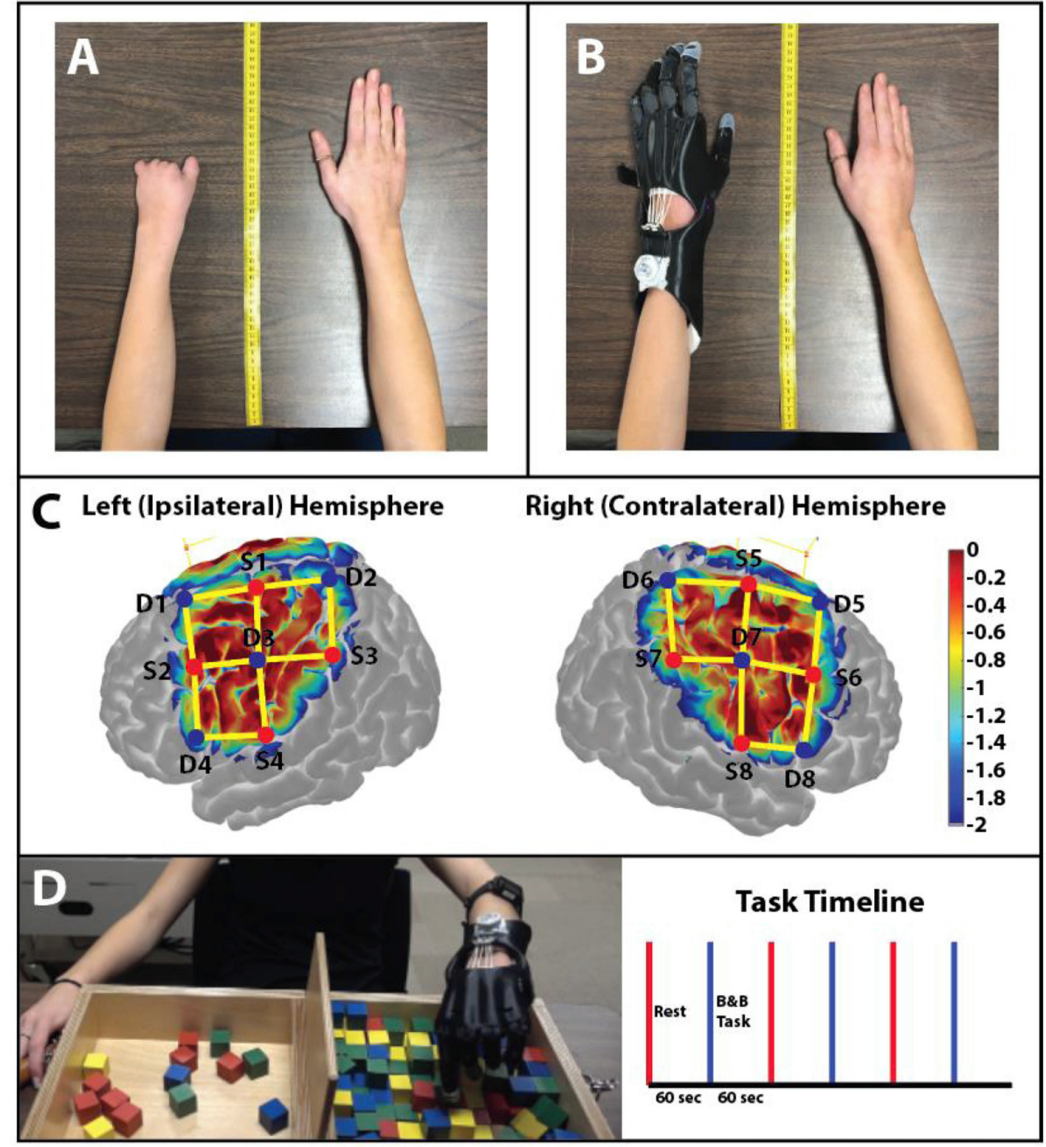
Anthropomorphic measurement photos. **(A)** Photo of the participant's non-affected (right) and affected (left) hand without the prosthetic hand. Measurements from the upper limbs were used to create a custom 3D printed prosthesis. **(B)** The 3D printed prosthetic hand used by the participant in this study. The prosthetic hand was modeled from the original design of the Cyborg Beast. **(C)** Sensitivity profile of the probe (Left and Right Hemispheres shown) used in this study. Log sensitivity index values closer to zero (red) reflect greater sensitivity for acquiring signals in that region (Aasted et al., 2015). Image created using AtlasViewer (v2.12.4). The location of the detectors (blue), the sources (red), and the channels (yellow lines) are shown for the subject. (**D)** B&B Task: Protocol of the study: 60 s of rest (red) followed by 60 s of the B&B task (blue). A total of 3 trials were conducted for the affected hand.

The subject evaluated was a 19-year-old female with a unilateral carpus upper limb reduction (ULD) of the left hand (Figure 1A). The participant was asked to visit the laboratory on three occasions. During the first visit (i.e., introductory), an overview of the study was explained to the participant and informed consent was obtained. During the second visit (i.e., baseline), the prosthesis was fitted (Figure 1B) and baseline testing was conducted. After 8 weeks of home intervention training with the 3D printed hand prosthesis (i.e., follow-up), the participant repeated all assessments. The study was approved by the University of Nebraska at Omaha Review Board.

### Isometric Testing & Electromyography (EMG) Recordings

Maximal voluntary isometric contraction (MVIC) was measured during wrist flexion and extension using a muscle testing dynamometer (microFET3, Hoggan Health Industries, West Jordan, UT). EMG signals were simultaneously recorded with two separate wireless bipolar surface electrodes (Avanti Trigno, Delsys, Natick, MA) with a sampling rate of 2,148 Hz, bandwidth of 20-450 Hz, common mode rejection ratio >80 dB, and noise <0.75 μV. Surface EMGs were placed over the flexor capri ulnaris and extensor digitorum muscles of the affected forearm as recommended from the SENIAM Project (Hermens et al., 1999). EMG signals were processed through EMGWorks Analysis (Delsys, Natick, MA), where the RMS of the signal was calculated. Data were normalized using EMG activity from 10 s MVIC of each muscle. During the MVIC test, participants were instructed to exert maximal effort and perform to the best of their ability. Data were normalized using RMS values during the middle 8 s of each 10 s MVIC. Co-contraction was calculated by the Coactivation index (CI). The middle 2 s of the contraction are used to calculate the coactivation index (Ervilha et al., 2012; Zuniga et al., 2018).

$$\begin{array}{l} CI=\;\frac{EMG\;Antagonist}{EMG\;Agonist}*100 \end{array}$$

### 3D Printed Partial Hand Prosthesis

The prosthesis was designed utilizing the measurements of the participant's affected and non-affected hand to create a symmetrically designed prosthetic hand (Figure 1B) (Zuniga et al., 2019b). Elastic cords provide passive finger extension. Finger flexion is driven by non-elastic cords and is activated through 20–30° of wrist flexion. The result is a composite fist (flexing the fingers toward the palm) for pinch grasp. The files for the design are available online on the National Institutes of Health (NIH) 3D print exchange website (Zuniga et al., 2014, 2015) and Thingiverse (Zuniga et al., 2014).

### Functional Near-Infrared Spectroscopy (fNIRS)

Data were collected using a continuous wave fNIRS system (NIRSport 2, NIRx Medical Technologies, LLC, Berlin, Germany). Data was sampled at 8 Hz operating at 760 and 850 nm wavelengths. The cap held eight sources and eight detectors (~3 cm distance from the source “long separation detectors”) with no short separation detectors. The cap was positioned on the head following the 10–20 international system (Klem et al., 1999) (Figure 1C). The fNIRS channels covered the area around the C3 and C4 landmarks which have been shown to detect motor activity that drives hand and arm movement (Nishiyori et al., 2016).

### Home Intervention Training

The participant was asked to perform modified activities from the Goal-Oriented Assessment of Lifeskills (GOAL) used to assess the performance of activities of daily living over a period of 8 weeks of prosthetic use (2 times per week) (Miller et al., 2013). The participant completed timed activities to assess functional completion of activities of daily living with the prosthesis under the direction of trained study personnel. All activities were timed by telling the participant, “Ready, Set, Go” and ending with “Stop” with the exception of the drawing activity. The goal for each task was to complete the task even if error occurred during the task. Each task was timed from start to completion. The participant completed these activities at least twice a week with a team member via webcam to collect the data. The training activities included Utensils, Paper Activities, Tray Carry, and Ball Play. Block Building and Bike Circuit were only conducted before and after the intervention and served as non-training tasks.

Utensils consists of using the participant's non-affected extremity and the prosthetic appendage to make four cuts in molding putty with a fork and knife. The fork was stabilized with a universal cuff attached to the prosthesis. The knife was used with the non-affected hand.

Paper Activities consists of several parts, stabilizing a piece of paper while the participant draws on it which was untimed, folding the piece of paper, stabilizing a tape dispenser with the prosthesis to tear 3 pieces of tape, and holding the paper with the prosthesis while the able hand uses scissors to make 3 cuts.

In Ball Play the participant must use the prosthesis to pick up a ball from the ground and either underhand or overhand toss it to a partner 3 times in a row.

Tray Carry has participants walk in a figure-8 formation around two toys placed ~5 feet apart while balancing a tray loaded with two cups with their prostheses and non-affected upper extremity.

### Non-training Tasks

The Box and Block (B&B) Test (Figure 1D) has been suggested as a measure of unilateral gross manual dexterity (Mathiowetz et al., 1985, 1986) and has been previously used to assess upper-limb prosthetic performance and motor learning (Mathiowetz et al., 1986; Dromerick et al., 2008; Young et al., 2019; Zuniga et al., 2019a,b). The B&B task was performed during the first and last week of the home intervention.

The Block Building activities included three trials of a series of 6 different block building activities for each hand separated by 30 s of rest (a total of 18 block building activities per hand). Specifically, the participant was instructed to perform three trials of the following building block activities: 4-block train (Block #1), 3-cube bridge (Block #2), 4-block wall (Block #3), 3-block tower (Block #4), 6-block steps (Block #5), and 6-block pyramid (Block #6). The Block Building activity was performed during the first and last week of the home intervention.

The Bike Circuit requires participants to either ride a bike or walk a bike in a figure eight around two stationary objects ~5 feet apart to demonstrate independent bilateral forearm activation in order to turn the bike with the able appendage and the prosthesis. The Bike Circuit was performed during the first and last week of the home intervention.

### Data Analysis and Statistics

**Figure 2:**
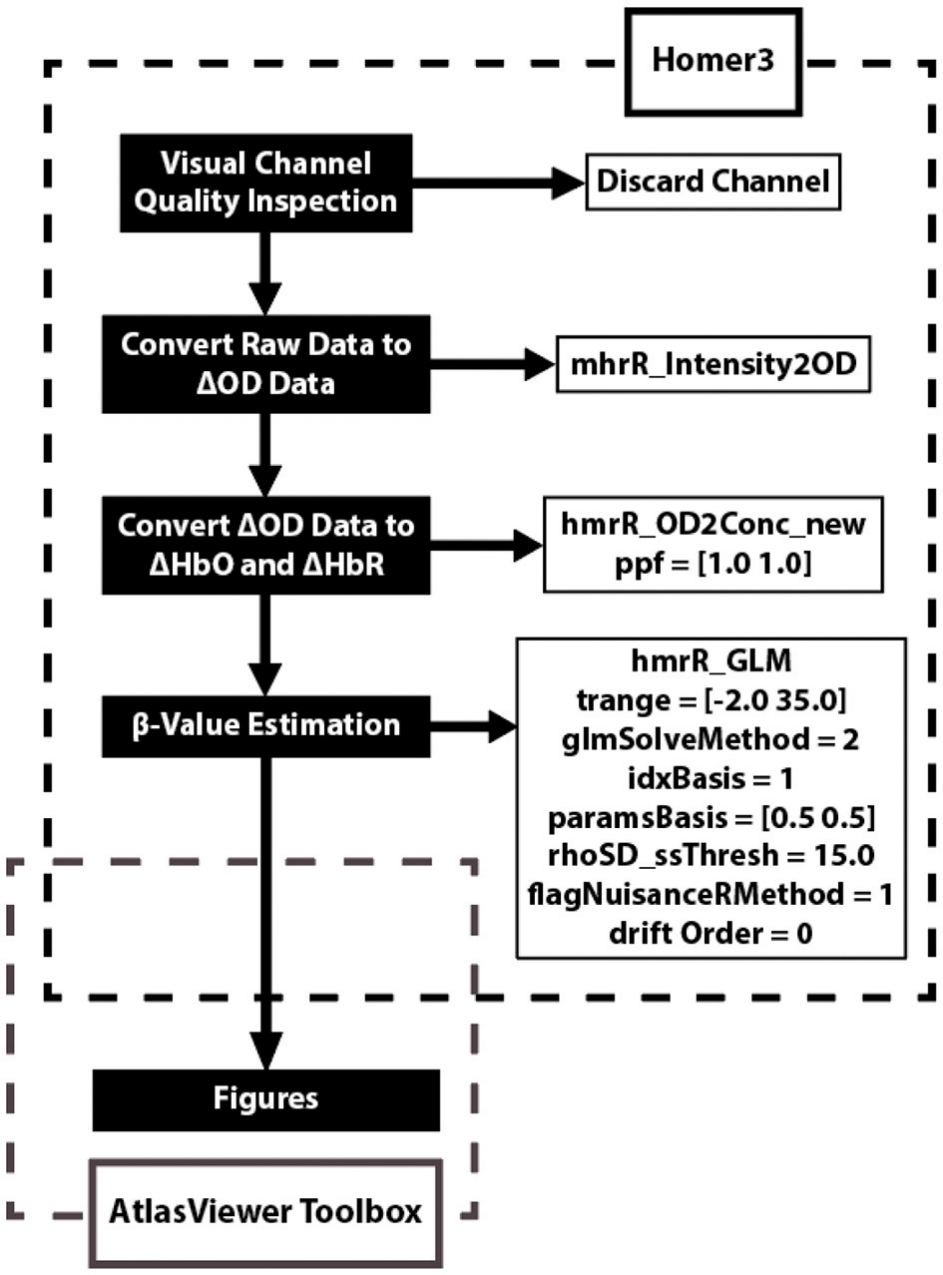
Data analysis flow chart applied to each fNIRS channel. The data analysis utilizes two open source toolboxes: Homer3 (v1.26) and AtlasViewer (v2.12.4) Toolboxes. The Homer3 toolbox provides a user-friendly graphic user interface (GUI) that allows for the creation of easy-to-use data analysis pipelines and figures. The functions and parameters used in Homer3 are provided in the dashed black box. First, raw fNIRS signals were converted into changes in optical density data by taking the logarithm of the signal. Second, the changes in oxygenated-hemoglobin (ΔHbO) concentrations were then obtained using the modified Beer-Lambert law with a partial pathlength factor of 1. Third, the hemodynamic response function (HRF) was then estimated by a general linear model (GLM) approach that uses iterative weighted least squares (Barker et al., 2013). Finally, figures were created using the AtlasViewer Toolbox, which provides an application for imaging and reconstruction of fNIRS data on atlas anatomy.

Data was analyzed using the Homer3 (v1.26) Toolbox (BUNPC, Huppert et al., 2009). Hemodynamic data were reconstructed on atlas anatomy utilizing the AtlasViewer (v2.12.4) Toolbox (Aasted et al., 2015). The data processing procedures are reported in Figure 2. The response was modeled using consecutive Gaussian temporal basis functions with a standard deviation of 0.5 s and with their means separated by 0.5 s over the regression time range of −2 to 35 s. This time period was chosen to include the full hemodynamic response to the task. β-values were calculated during the GLM analysis and represent the weighted responses of the individual channels during the task compared to baseline levels.

## Results

### Anthropomorphic & EMG Measurements

There was a notable increase in Range of Motion of the affected (wrist extension = 75° to 115°; wrist flexion = 70° to 120°) wrist after the home intervention training. Maximum voluntary contraction (MVC) increased during flexion (Baseline = 17.4 ± 0.6 lbs; Follow-Up = 39.0 ± 5.4 lbs). Conversely, MVC decreased during extension (Baseline = 33.7 ± 0.5 lbs; Follow-Up = 26.9 ± 2.1 lbs). The Coactivation Index (CI; no units) decreased during flexion (Baseline = 31.10 ± 1.54; Follow-Up = 23.11 ± 9.98) and increased during extension (Baseline = 17.33 ± 1.49; Follow-Up = 25.43 ± 1.94). Thus, the training may have increased wrist flexor tone in the affected limb.

### Home Intervention Training

**Figure 3:**
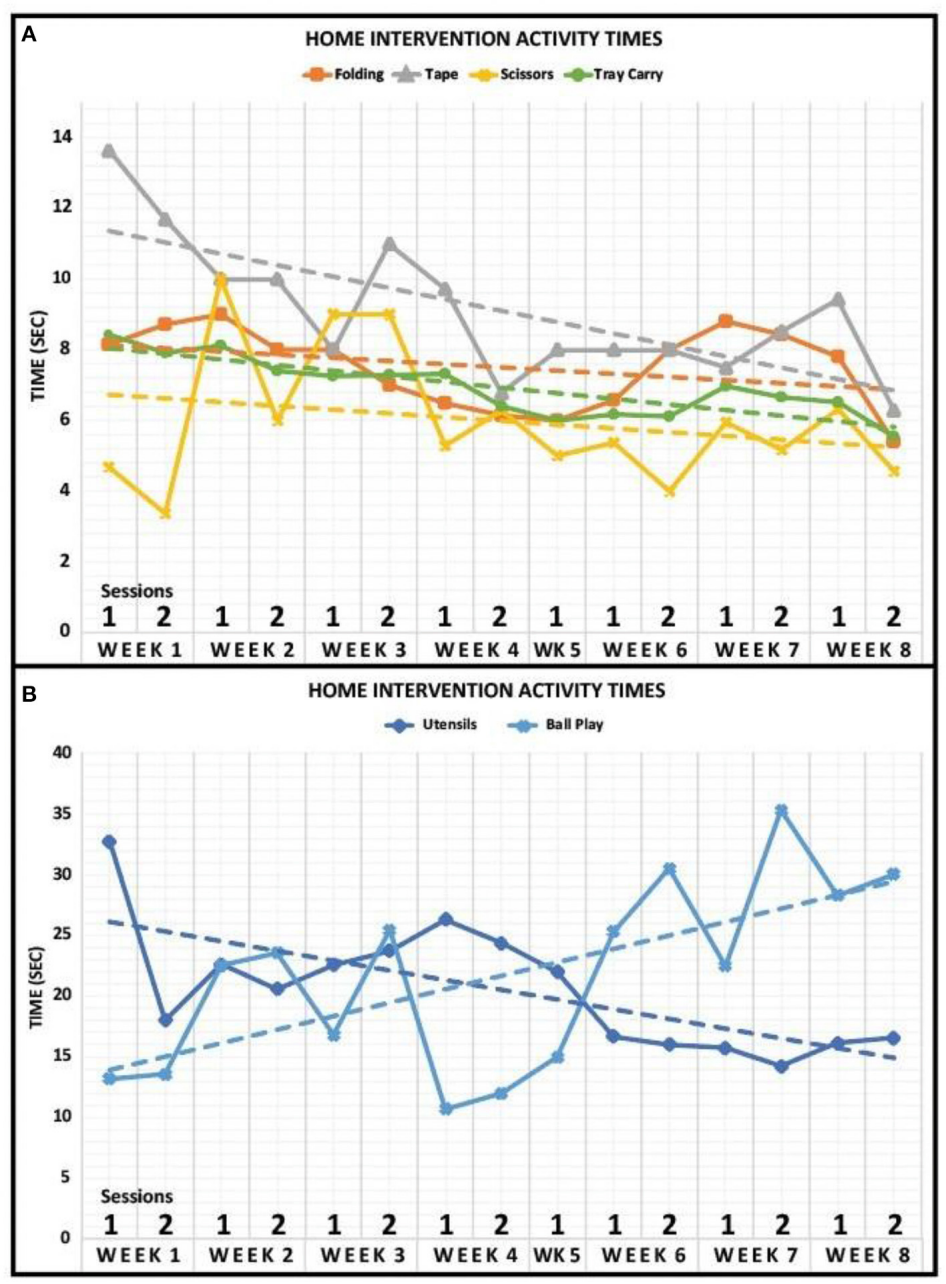
Home intervention activity times. **(A)** Activity times for Folding, Tape, Scissors, and Tray Carry are displayed for 8 weeks. The case participant completed both sessions for every week of the home intervention training except for week 5. Dashed lines represent a linear fit of the data. **(B)** Activity times for Utensils and Ball Play are displayed for 8 weeks. The case participant completed both sessions for every week of the home intervention training except for week 5. Dashed lines represent a linear fit of the data.

The activity times for each task of the home intervention training are displayed in Figures 3A,B. The data revealed that there was a negative trend in the amount of time to complete Utensils, Folding, Taping, Scissors, Tray Carry, and Bike Circuit activities over the course of the 8-week intervention period. The activity of Ball Play represented a positive trend in the time it took to complete across the 8-week intervention period.

The data for each block task revealed for the affected hand that there was a negative trend in the amount of time to complete Block Tasks #2 (Baseline = 8.38 s; Follow-Up = 5.11 s), #3 (Baseline = 9.35 s; Follow-Up = 8.18 s), and #4 (Baseline = 17.48 s; Follow-Up = 16.28 s) while there was a positive trend in the amount of time to complete Block Tasks #1 (Baseline = 5.70 s; Follow-Up = 11.62 s), #5 (Baseline = 20.85 s; Follow-Up = 46.41 s), and #6 (Baseline = 20.92 s; Follow-Up = 27.29 s). Overall, the average time to complete all block tasks increased after the home intervention training (Baseline = 13.78 s; Follow-Up = 19.15 s) for the affected hand. Additionally, the time to complete the Bike Circuit decreased after the home intervention training (Baseline = 20.17 s; Follow-Up 17.79 s).

### Gross Manual Dexterity Task

The case participant was able to move more blocks after the home intervention training (Baseline = 12.00 ± 3.46 blocks; Follow-Up = 19.33 ± 4.16 blocks), conducting the task at her own determined pace.

### Oxygenated Hemoglobin (HbO) Projections to Cortex

**Figure 4:**
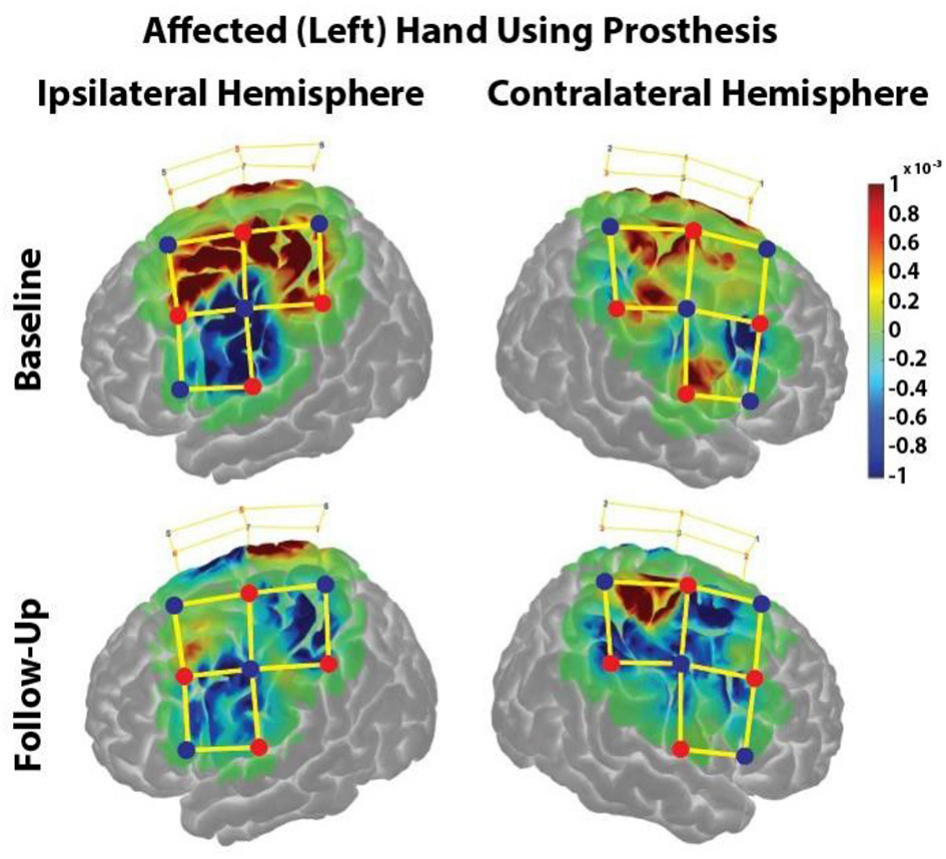
HbO responses during the Box & Block Task using the affected (left) hand before and after the home intervention training. Change in HbO is shown for each channel (yellow lines). An increased HbO response is highlighted in red while a decreased HbO response is highlighted in blue. The change in HbO is centered on zero, where a change in HbO greater than a threshold of *p* < 0.01 were plotted. The color bar indicates the scale of the concentration change on the logarithmic scale. Red dots are source optodes, and blue dots are detector optodes. Figures were created using AtlasViewer.

The HbO response during the Box & Block task was projected onto a brain model using AtlasViewer to further interpret specific brains regions of interest (Figure 4).

In the ipsilateral hemisphere during the Baseline visit, there was strong cortical activation in the medial regions of the premotor, primary motor, and somatosensory cortices (Figure 4, Upper Left). During the Follow-Up visit, there was minimal cortical activation in only the medial aspect of the premotor cortex (Figure 4, Lower Left).

In the contralateral hemisphere during the Baseline visit, cortical activation was restricted in the medial aspects of the somatosensory and primary motor cortices as well as the lateral aspect of the primary motor cortex (Figure 4, Upper Right). During the Follow-Up visit, there was strong cortical activation in in the medial primary and somatosensory cortices, (Figure 4, Lower Right).

## Discussion

In the current investigation, a case participant with a ULD of the left hand partook in an 8-week home intervention training while using a custom 3-D printed partial hand prosthesis. The primary finding of the current investigation is that an 8-week prosthetic home training was able to reverse abnormal brain lateralization in this young adult with congenital partial hand reduction by significantly increasing cortical activation in the contralateral hemisphere. Increased cortical activation was supported by an increased range of motion along with increased flexor tone of the affected limb; however, the extensor tone of the affected limb decreased. More importantly, time to complete activities of daily living trended downward over the 8-week home intervention training, except during block activities and ball play.

### Baseline Measurements

Baseline measurements of maximum voluntary isometric contraction (MVIC) during flexion agree with our previously reported measurements (Zuniga et al., 2018). However, the case participant presented with a higher MVIC measurement during extension, which caused a dissimilar comparison of measurements to our previous measurements (Zuniga et al., 2018). This difference is most likely due to the case participant being older in age.

Before training, there was a strong ipsilateral cortical response during a gross manual dexterity task while using a partial hand prosthesis, which was consistent with our previous findings (Zuniga et al., 2021). Additionally, this ipsilateral response seen before training agrees in adults with acquired amputation of the preferred right hand (Philip and Frey, 2014). In children, there is evidence of an ipsilateral functional reorganization after an upper limb amputation along with cortical disinhibition contralateral to the amputated area (Hamzei et al., 2001). In contrast, the case participant experiencing congenital limb loss showed minimal and non-focalized increases in cortical activity in the contralateral hemisphere before training. With this minimal contralateral cortical response, the case participant thus showed minimal cortical coactivation of the contralateral hemisphere, which disagrees with the findings of Hamzei et al.; however, this difference can be due to congenital versus acquired origin of the limb reduction. Thus, it is conceivable that the participant with ULD is less susceptible to contralateral cortical disinhibition since her hand region was deprived due to the absence of the hand at birth (Makin et al., 2013).

### Use-Dependent Training of the Affected Limb

Use-dependent plasticity, which describes neural changes as a result of the repeated practice of movements, has been demonstrated in the human cortex (Classen et al., 1998; Koeneke et al., 2006; Raffin and Siebner, 2019). Classen et al. demonstrated that a short training period, consisting of simple, voluntary, repetitive thumb movements in a specific direction, elicits reorganization of the cortical representation of the thumb. The partial hand prosthesis was a body-powered device activated by wrist flexion, which causes the hand to close in a gross, grasping motion. This grasping motion allowed the participant to grasp and pinch objects. As a result of the training, the case participant showed various improved performances as shown in increased flexor tone, as reported by increased maximum voluntary isometric contractions, as well as decreases in home intervention task times and in various blocks tasks. Unexpectedly, the extensor tone decreased after intervention, which does not agree with our previous study except for one subject (Zuniga et al., 2018). Since the flexors are extensively used during the intervention, future studies will need to observe the extensor use during the intervention to determine any lack-of-use reasoning for this decrease in extensor tone or if atrophy over time is common with this congenital disorder.

Although the time to complete the block tasks increased overall while using the partial hand prosthesis, it was noted that the case participant stacked the blocks with the partial hand prosthesis in a more organized and coordinated manner which resulted in an increased time. Increased time also was due to error like knocking over the blocks while stacking. However, since the block tasks were not performed each week of the home intervention therapy (only one session before and after training), the increased task time could indicate that tasks not performed in a training procedure do not become learned. Future studies will need to record, or exclude, errors that occur during the tasks performed as well as qualitatively or quantitatively measure any improvements in coordination that may occur while using the prosthesis. Furthermore, increased flexor tone and continued use of the partial hand prosthesis improved gross hand dexterity as shown in the increased number of blocks moved during the gross manual dexterity task. These improvements are similar to previous investigations that have shown improvements in the range of motion of the affected wrist and forearm circumference (Zuniga et al., 2016), as well as case reports indicating improvements in function (Zuniga et al., 2015, 2019a) after using 3-D printed transitional prostheses. Likewise, the change in cortical response shown in this study are supported by similar studies reporting use-dependent plasticity in the contralateral, corticomotor region after training (Koeneke et al., 2006; Raffin and Siebner, 2019).

Hahamy et al. showed that compensatory behaviors utilized the de-afferented area contralateral to the missing hand (Hahamy et al., 2017). After training, the contralateral activity became more centralized to the primary motor cortex, most likely a representation of the increased flexor tone and improved performance during the B&B task. Additional changes in cortical activity could be indicative of compensatory strategies to use the prosthetic device (i.e., compensatory muscles used to guide the prosthesis). This centralized, contralateral activity in unison with a significantly decreased cortical response in the ipsilateral hemisphere, indicating a reduced use of the ipsilateral hemisphere. It has been reported that use-dependent plasticity reveals a synergistic rather than competitive interaction between hemispheres (Raffin and Siebner, 2019). Future studies will need to measure the interconnectivity between hemispheres as well as use of the non-affected hand to identify the reason for the decreased activity in the ipsilateral hemisphere.

## Conclusion

The current investigation described the changes in cortical activation measured by oxygenated hemodynamic (HbO) responses and improved performances that resulted from an 8-week home intervention training while using a custom 3D printed partial hand prosthesis in a case participant with a congenital upper-limb reduction. More importantly, the results of the current case study suggest that the home intervention training was able to reverse abnormal brain lateralization in this young adult with congenital partial hand reduction by significantly increasing cortical activation in the contralateral hemisphere. These changes in cortical activation and improved performances followed the mechanisms underlying use-dependent plasticity. The results from this case participant provides qualitative evidence of use-dependent plasticity in individuals with congenital limb reductions. This knowledge will assist to identify if the current reversed abnormal brain lateralization results in an increased prosthetic acceptance rate in a larger group of individuals with congenital limb reduction.

## Data Availability Statement

The raw data supporting the conclusions of this article will be made available by the authors, without undue reservation.

## Ethics Statement

The studies involving human participants were reviewed and approved by The University of Nebraska at Omaha Review Board. The patients/participants provided their written informed consent to participate in this study. Written informed consent was obtained from the individual(s) for the publication of any potentially identifiable images or data included in this article.

## Author Contributions

JZ designed the study and the prosthesis, and is the Principal Investigator of the study. JB wrote the majority of the manuscript. JB, JZ, CC, JL, and KF collected the data and tested the prototypes. JB and JZ edited the manuscript. JB conducted the fNIRS analysis, wrote the code for the analysis, and interpreted the results. CC analysed the EMG data. JL and KF conducted the home intervention training, and JL provided the home training data. All authors read and approved the final manuscript.

## Conflict of Interest

JZ, Ph.D. is the designer of the 3D printed prostheses Cyborg Beast. The remaining authors declare that the research was conducted in the absence of any commercial or financial relationships that could be construed as a potential conflict of interest.
